# Psychoacoustic and electroencephalographic responses to changes in amplitude modulation depth and frequency in relation to speech recognition in cochlear implantees

**DOI:** 10.1038/s41598-024-58225-1

**Published:** 2024-04-08

**Authors:** Nina Aldag, Waldo Nogueira

**Affiliations:** Department of Otolaryngology, Hannover Medical School and Cluster of Excellence ‘Hearing4all’, Hanover, Germany

**Keywords:** Biomedical engineering, Cortex, Acoustics, Electrical and electronic engineering

## Abstract

Temporal envelope modulations (TEMs) are one of the most important features that cochlear implant (CI) users rely on to understand speech. Electroencephalographic assessment of TEM encoding could help clinicians to predict speech recognition more objectively, even in patients unable to provide active feedback. The acoustic change complex (ACC) and the auditory steady-state response (ASSR) evoked by low-frequency amplitude-modulated pulse trains can be used to assess TEM encoding with electrical stimulation of individual CI electrodes. In this study, we focused on amplitude modulation detection (AMD) and amplitude modulation frequency discrimination (AMFD) with stimulation of a basal versus an apical electrode. In twelve adult CI users, we (a) assessed behavioral AMFD thresholds and (b) recorded cortical auditory evoked potentials (CAEPs), AMD-ACC, AMFD-ACC, and ASSR in a combined 3-stimulus paradigm. We found that the electrophysiological responses were significantly higher for apical than for basal stimulation. Peak amplitudes of AMFD-ACC were small and (therefore) did not correlate with speech-in-noise recognition. We found significant correlations between speech-in-noise recognition and (a) behavioral AMFD thresholds and (b) AMD-ACC peak amplitudes. AMD and AMFD hold potential to develop a clinically applicable tool for assessing TEM encoding to predict speech recognition in CI users.

## Introduction

The cochlear implant (CI) is probably the most successful prosthetic device in modern times, enabling people with profound hearing loss to understand speech^1,2^. CIs are successful in transmitting slow temporal fluctuations of speech, and the ability to discriminate temporal envelope modulations (TEMs) in different frequency bands has been related to speech recognition^3–6^. However, there is large variability in performance outcomes among CI users. Some of the known factors contributing to the outcome variability are demographic factors such as age^7^, duration of deafness^8^, and age at implantation^7^, surgical factors such as CI electrode placement^9^, and device specific factors such as electrode design^10^ and speech-processing strategy^10^. Moreover, the CI requires the fitting of several parameters that are set based on behavioral measures of sound perception. The result of the fitting is then verified by standardized speech recognition tests. In addition, CI fitting is time-consuming and subjective as it depends on technical personnel and the behavioral response of the CI user^1^. This study investigates a method to assess the hearing performance of CI users based on TEMs. The method examines central potentials using electroencephalography (EEG), making it more objective than traditional speech recognition tests. Additionally, the stimuli used can be directly linked to channel-specific parameters, thus providing a link to the clinical CI fitting.

The envelope of a speech signal contains amplitude modulations between 2 and 50 Hz and can primarily convey information about the manner of articulation, tempo, rhythm and the syllabicity^11^. Shannon et al.^5^ showed that a good speech understanding can be based primarily on such temporal cues. The signal processing strategy of the CI computes a very simplified representation of speech, mostly based on multiband TEMs with a maximum modulation frequency of 400 Hz^12^ delivered by an electrode array in the cochlea. TEMs can be characterized by the depth and by the frequency of the amplitude modulation. Amplitude modulation detection (AMD) describes the ability to perceive amplitude changes in the temporal envelope. Amplitude modulation frequency discrimination (AMFD) describes the ability to distinguish between different modulation rates in the temporal envelope. The good speech recognition of CI users, despite the degraded information transmission, suggests that TEM encoding plays an important role in conveying speech information through the CI. Several studies have demonstrated that TEM encoding abilities are strongly related to speech recognition in CI users. For example, Fu^13^ showed that behaviorally assessed AMD thresholds correlate with phoneme recognition. In addition, Luo et al.^14^ showed that AMD thresholds and AMFD thresholds were correlated to several speech perception measures in Mandarin-speaking CI users. Erb et al.^15^ predicted 6-month speech understanding from postoperative AMFD thresholds and suggested that AMFD thresholds could be a clinical measure of temporal resolution. In summary, AMD and AMFD thresholds have the potential to be incorporated into clinical practice for performance assessment during the regular CI check-ups. However, the transition of such a measurement into the clinic has failed so far, probably because of the lengthy and cumbersome testing procedure required to investigate behavioral AMD and AMFD thresholds. This problem could be overcome by electrophysiological measures that do not require the active feedback from the patient, making the test more objective and less burdensome for the patient and the audiologist.

In modern CI clinics, the only electrophysiological measures routinely used are the acoustic reflex threshold (ART), electrocochleography (ECochG) responses, the electrically evoked compound action potential (eCAP), and the electrically evoked auditory brainstem response (eABR). These peripheral measures have been shown to be useful for diagnosis, for the evaluation of the electrode-nerve interface^16^, and in the fitting of CI parameters^17^. However, the correlation between eCAPs, eABRs and behavioral thresholds is only moderate, probably due to the mismatch between stimuli to derive behavioral threshold or comfort level (T-, C-level) and to derive eCAPs and eABRs^18^. In addition, these peripheral measures do not relate to speech recognition because the auditory periphery does not fully characterize the auditory pathway and differences from the brainstem to the auditory cortex as well as cognitive aspects may lead to differences in speech recognition^19^. Event-related potentials (ERPs) are a useful and clinically applicable measure of the central auditory pathway using EEG. The cortical auditory evoked potential (CAEP) is a neurophysiological correlate of auditory stimulus detection that can be used, for example, to estimate the threshold level (T-level) through amplitude growth functions^18,20^.

Special variants of the CAEP are the mismatch response^21^ (MMR) and the acoustic chance complex^22^ (ACC). Both responses are measures of auditory discrimination. The MMR is evoked in an oddball paradigm and typically includes the mismatch negativity^23^ (MMN) and the P300^24^ in adults. However, the current clinical relevance of the MMR is limited by its small amplitude^25^ and the fact that assessment of the P300 requires active patient participation. The ACC is a transient response that is elicited by changes in a continuous stimulus. The ACC has good test–retest reliability, can be recorded in special populations such as in children and CI users, and shows good agreement with behavioral measures^26^. An important side note is that the above mentioned EEG responses can be recorded in CI users. However, the EEG of CI users is highly contaminated by the electrical artifact, which must be appropriately treated in post-processing to ensure that the physiological response is not confused with the CI artifact.

Studies have shown that the ACC can be used to assess TEM encoding in CI users. For example, the ACC can be elicited by a change in modulation depth^27^ or in modulation frequency^28,29^. The degree of change increases the amplitude of the ACC^26^, making it a valuable tool for objectively assessing AMD and AMFD abilities. Therefore, AMD-ACCs and AMFD-ACCs could be used as clinical measures of temporal resolution and speech recognition in CI users. Han and Dimitrijevic^30^ previously showed that AMD-ACCs at 40 Hz were correlated with vowel, consonant, word and sentence recognition in quiet and in noise. However, AMD can be highly confounded by loudness cues, requiring a loudness balancing procedure prior to the measurement^31^. Loudness balancing is not required for AMFD, since loudness is almost constant across different modulation frequencies^32–34^. To our knowledge, no study has related AMFD-ACCs to speech recognition.

Another electrophysiological measure of TEM encoding that has received increasing attention is the auditory steady-state response (ASSR). The ASSR is an ongoing response that is elicited by periodic changes in a continuous stimulus. It is represented by oscillations at the frequency of the periodic changes in the stimulus. Measuring ASSRs in CI users is challenging because the CI stimulus artifact is difficult to separate from the neural response^35^. However, Hofmann and Wouters^35^ proposed a strategy for CI artifact removal that allows recording of ASSRs in CI users. In a follow-up study the ASSR thresholds correlated with behaviorally assessed T-levels^36^. In addition, Gransier et al.^37^ showed that the variability of the ASSR across CI electrodes was correlated with speech-in-noise perception.

In this study, we investigate a new EEG paradigm that combines different measures of TEM encoding in CI users. The goal was to find the best correlate between electrophysiological and behavioral measures of TEM encoding and speech recognition. We combined the measurement of AMD-ACCs, AMFD-ACCs, and the ASSR to amplitude modulated pulse trains in a single 3-stimulus paradigm studied with a clinical EEG system. The results of this paradigm were compared with a 2-stimulus alternating paradigm shown by Undurraga et al.^28^. Direct electrical stimulation at individual CI electrodes was used to ensure the correspondence between CI fitting and outcome measures. Stimulus parameters were varied to examine differences between basal and apical stimulation and differences between modulation frequencies. The hypotheses of this study were that (1) this novel 3-stimulus paradigm would allow us to simultaneously record the onset CAEP, the AMD-ACC, the AMFD-ACC, and the offset CAEP, (2) we could additionally measure ASSRs during the amplitude modulated stimuli using a single basal or apical electrode for stimulation and state-of-the-art artifact-rejection strategies, (3) we would observe consistent results between behaviorally assessed TEM encoding and the ACC results, (4) the behaviorally assessed AMFD would correlate with speech recognition, and (5) the AMD-ACCs and the AMFD-ACCs could be used as predictors of speech recognition.

## Results

### Behavioral data

**Figure 1:**
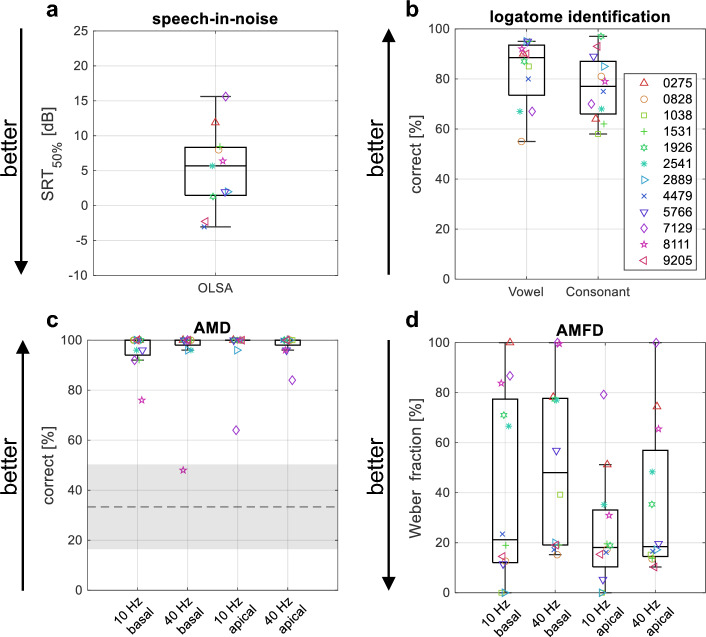
Behavioral test results. Each subject is represented by a unique marker symbol and color. (**a**) Speech-in-noise recognition as measured by the Oldenburg Sentence Test (OLSA). The speech reception threshold (SRT_50%_) marks the signal-to-noise ratio at which 50% of the words are understood. Note that subject 1038 could not complete the OLSA because the subject exceeded the maximum SRT_50%_ of 25 dB; (**b**) Vowel and consonant identification test results as the percentage of correct responses; (**c**) Behavioral responses for the amplitude modulation detection (AMD) task as the percentage of correct responses. The shaded area is the chance level around the guessing rate (dashed line); (**d**) Behavioral responses for the amplitude modulation frequency discrimination (AMFD) task.

Subjects achieved a median speech reception threshold (SRT_50%_) of 5.7 dB (range: − 3.0 dB to 15.6 dB). Subject 1038 could not complete the Oldenburg Sentence Test (OLSA) because the maximum SRT_50%_ of 25 dB was exceeded, so this subject was excluded from all analyses comparing the speech-in-noise performance to other measures. The median vowel identification score across subjects was 88.5% (range: 55% to 95%). The median consonant identification score was 77% (range: 58% to 97%). The speech test results for all subjects are shown in Fig. 1a,b. There was no significant correlation between the different speech test scores.

The AMD test results shown in Fig. 1c are above chance level (50.3%), as determined by a binomial distribution around the guessing rate (1/3) with a 5% confidence interval, for all subjects in all conditions except for subject 8111 in the detection of 40 Hz modulation with apical stimulation. Almost all subjects were able to detect the 100% modulation. The median percentage of correctly identified trials was 100% for all four conditions. Friedman’s two-way ANOVA test showed that there was no significant effect for the factor stimulation electrode (p = 0.360) or for the factor modulation frequency (p = 0.693).

The AMFD test results are shown in Fig. 1d. The Weber fraction is distributed between 0%, indicating that all modulation frequencies tested were discriminated by the subject, and 100%, indicating that none of the modulation frequencies tested were discriminated. The median Weber fractions were 21.2%, 48.0%, 18.1%, and 18.4% for the 10 Hz basal, 40 Hz basal, 10 Hz apical, and 40 Hz apical conditions, respectively. Friedman’s two-way ANOVA test showed that there was no significant effect for the factor stimulation electrode (p = 0.106) or for the factor modulation frequency (p = 0.198).

### Electrophysiological data

#### Acoustic change complex

**Figure 2:**
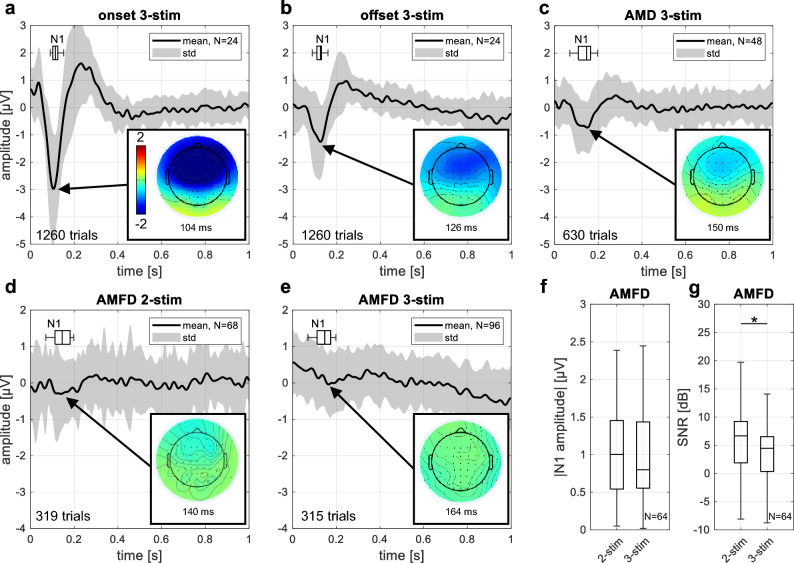
Event related potentials. (**a**–**e**) Plot of mean cortical responses at channel FCz. The mean across subjects and conditions is shown in bold black and the standard deviation is shown in gray. The number of trials included in the average for each subject is noted in the lower left corner. The N1 latencies of individual subjects are shown with the horizontal box plot in the upper left. Topographic plots of cortical responses show the average voltage spread across all subjects at different electrode locations in the axial plane, with the nose at the top. Negative voltage is colored blue, positive voltage is colored red. The latency corresponds to the latency of the N1 peak in the averaged curve. (**a**) Onset response (pause–S1); (**b**) Offset response (S3–pause); (**c**) Acoustic change complex (ACC) for amplitude modulation detection (AMD, S1–S2); (**d**,**e**) ACC for amplitude modulation frequency discrimination (AMFD, S2–S3) for 3-stimulus paradigm (**d**) and for 2-stimulus paradigm (**e**); (**f**,**g**) Comparison between AMFD 2- and 3-stimulus paradigms regarding the absolute N1 peak amplitudes (**f**) and the signal-to-noise ratio (SNR) (**g**). Stars indicate significance differences between the 2- and the 3-stimulus paradigm (*p < 0.05).

The first hypothesis of this study was that we could measure four different ERPs with the novel 3-stimulus paradigm: Onset CAEP, offset CAEP, AMD-ACC, and AMFD-ACC. A visualization of the paradigm, including the expected ERPs, can be found in Fig. 8 in Section "Methods". For each response type, we analyzed which electrode position resulted in the highest overall N1 peak SNR across subjects. We found that for all ERPs, the highest SNR was located at electrode FCz (mean: 21.9 dB, 15.3 dB, 12.3 dB, 4.5 dB for onset, offset, AMD-ACC, and AMFD-ACC, respectively). We also tested whether the averaged signal from five fronto-central electrodes (Fz, FC1, FCz, FC2, and Cz) improved the responses and found that the averaged signal decreased the N1 peak amplitude for all ERPs. Based on this analysis, we decided to use electrode FCz for all subsequent ERP analyses. The four different ERPs are shown in Fig. 2a–c,e. Note that the different number of trials recorded for the four different ERPs may partially explain a lower SNR for the ACCs compared to the onset and offset responses, but not the smaller amplitudes.

In the novel 3-stimulus paradigm, the onset CAEP, the offset CAEP and the AMD-ACC could be reliably measured. The expected morphology of a negative peak (N1) at around 100 ms relative to stimulus onset and a positive peak (P2) later than 200 ms was visible in the averaged time curves. The topographies showed a negative activation in fronto-central areas. The N1 peak amplitude of the onset response was significant in 23 out of 24 measurements (96%), the offset response in 19 out of 24 measurements (79%), and the AMD-ACC in 37 out of 48 measurements (77%) (see Section "Data analysis" for details on the statistics). However, the N1 peak for AMFD-ACC in the 3-stimulus paradigm was not reliable. Only 31 out of 96 measurements (32%) showed a significant N1 peak, and the averaged topography did not show a negative activation in fronto-central areas (Fig. 2e).

Because the AMFD-ACC in the 3-stimulus paradigm was not as reliable as would be expected from the related 2-stimulus paradigm of Undurraga et al.^28^, we compared the AMFD-ACC from our novel 3-stimulus paradigm with a replication of the related 2-stimulus paradigm^28^ (Fig. 2d). In the 2-stimulus paradigm, the morphology of the averaged time course showed a broad negative peak from 115 to 215 ms, and the topography showed a negative activation in fronto-central areas. The median N1 amplitude was higher in the 2-stimulus paradigm, but the difference was not significant (Wilcoxon signed rank test: p = 0.411). However, the difference in signal-to-noise ratio (SNR) between the two conditions was significant (Wilcoxon signed rank test: p = 0.023), resulting in a higher number of significant N1 responses in the 2-stimulus than in the 3-stimulus paradigm (55% vs. 33%) when comparing the subjects who participated in all conditions (Fig. 2f,g). This result suggests that the 2-stimulus paradigm evoked slightly higher and more reliable responses. For this reason, we used the results of the 2-stimulus paradigm for all further evaluation of AMFD-ACC.

Overall, the median N1 amplitude across all subjects and all conditions was − 2.8 µV (interquartile range (IQR): 3.2 µV), − 1.3 µV (IQR: 1.6 µV), − 1.5 µV (IQR: 1.0 µV), and − 1.0 µV (IQR: 0.9 µV) for the onset response, offset response, AMD-ACC, and AMFD-ACC, respectively. The median N1 latency across all subjects and all conditions was 114 ms (IQR: 23 ms), 125 ms (IQR: 21 ms), 147 ms (IQR: 56 ms), and 143 ms (IQR: 72 ms), respectively.

#### Auditory steady-state response

**Figure 3:**
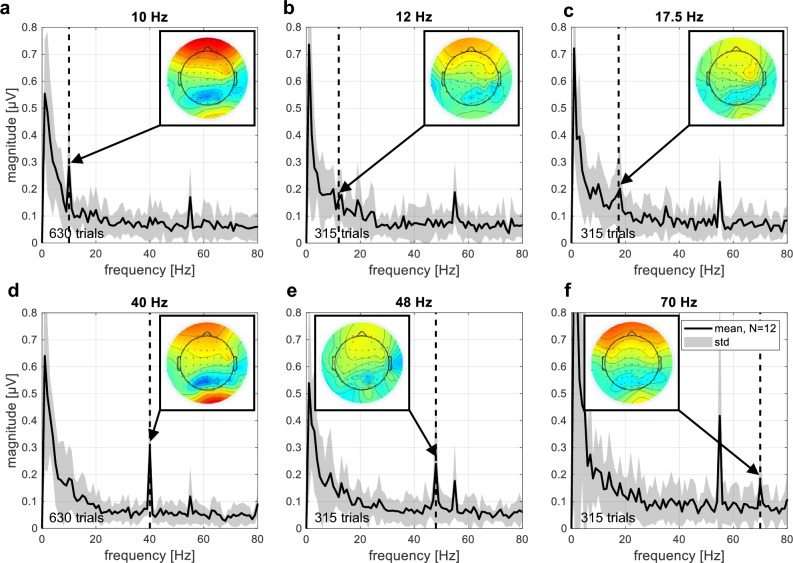
Auditory steady-state response (ASSR) for stimulation with an apical electrode at the modulation frequencies (**a**) 10 Hz; (**b**) 12 Hz; (**c**) 17.5 Hz; (**d**) 40 Hz; (**e**) 48 Hz; (**f**) 70 Hz at the electrode position FCz. The mean magnitude across subjects is shown in bold black and the standard deviation is shown in gray. The dotted vertical line marks the modulation frequency of the stimulus. The number of trials included in the average for each subject is noted in the lower left corner. Topographic plots of the ASSR show the average voltage spread across all subjects at different electrode locations in the axial plane, with the nose at the top. The relative power in dB is color-coded, with red representing the highest power and blue representing the lowest power.

Our second hypothesis was that ASSRs could be recorded with the novel 3-stimulus paradigm. The ASSR results for all modulation frequencies used in the experiment are shown in Fig. 3. Note that we only refer to the results for the stimulation with an apical electrode, the results for the stimulation with a basal electrode can be found in Supplementary Fig. A1. We analyzed which electrode position resulted in the highest overall peak SNR at all AM frequencies across subjects. Again, the highest SNR was found at electrode FCz (mean: 18.4 dB), and based on this analysis, we decided to use electrode FCz for all subsequent ASSR analyses. In general, the peak magnitude was higher with apical than with basal stimulation (0.23 µV and 0.15 µV, respectively), and the SNR was also higher with apical than with basal stimulation (12.9 dB and 8.1 dB, respectively). The spectrum showed distinct peaks at the modulation frequencies 10 Hz and 40 Hz of about 0.3 µV. The peaks at 12 Hz, 17.5 Hz, 48 Hz and 70 Hz had smaller magnitudes (0.17–0.26 µV), but were still visible in the average spectrum. Although the average magnitude between the 10 Hz and the 40 Hz ASSRs was almost the same, the SNR of the 40 Hz ASSR was higher compared to the 10 Hz ASSR (6.9 dB and 18.3 dB, respectively). For the other modulation frequencies, the mean peak SNR was 0.1 dB, 4.1 dB, 14.4 dB, and 9.8 dB for 12 Hz, 17.5 Hz, 48 Hz, and 70 Hz, respectively.

Note that there was also a distinct peak at 55 Hz in all stimulation conditions, including the unmodulated condition and also the pause between S3 and S1 (see Supplementary Fig. A2). During the pause, the CI was not stimulating, but “power up frames” were transmitted over the radio-frequency link to power the implant. Since the topographic location of the 55 Hz peak was located at the position of the powered implant (see Supplementary Fig. A2) and the peak was absent when the CI was turned off, we conclude that the peak was caused by CI artifact from the radio-frequency link in combination with under sampling at the 20 kHz EEG sampling rate.

### Relation between behavioral and electrophysiological measures

#### Comparison between behavioral and electrophysiological amplitude modulation data

**Figure 4:**
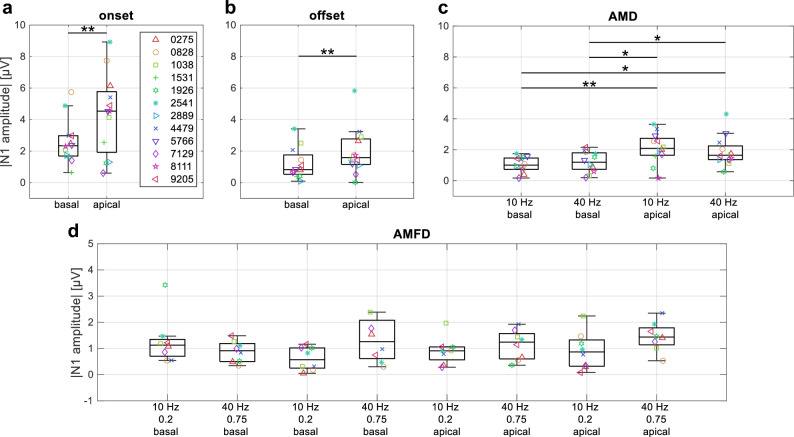
Comparison of apical and basal stimulation. The box plots show the absolute N1 amplitude for each condition. Each subject is represented by a unique marker symbol and color. (**a**) Onset response (pause–S1); (**b**) Offset response (S3–pause); (**c**) Acoustic change complex (ACC) for amplitude modulation detection (AMD, S1–S2); (**d**) ACC for amplitude modulation frequency discrimination (AMFD, S2–S3) in the 2-stimulus paradigm. Stars indicate significant differences (*p < 0.05; **p < 0.01) as tested between apical and basal stimulation after correction for multiple comparison (number of comparisons: n = 1 (**a**,**b**); n = 4 (**c**)). Note that in the later configuration (**d**), not all subjects participated in all test configurations (see Section "Data collection"). No significant effect was found when subjects with missing configurations were excluded from the test. All configurations are listed in Supplementary Table A2.

The third hypothesis was that behaviorally and electrophysiologically measured AMD-ACC and AMFD-ACC are related to each other. The difference between basal and apical stimulation electrode was investigated first. Based on previous studies^20,38,39^, it was expected that the absolute amplitude of the CAEPs and ACCs would be lower for basal than for apical stimulation. A comparison of apical and basal stimulation for the different conditions is shown in Fig. 4.

In general, the absolute N1 amplitude was higher for the apical than for the basal stimulation electrode. The Wilcoxon signed-rank test showed that the difference in N1 amplitude between apical and basal stimulation was significant for the onset and offset responses (both p = 0.007). For the AMD data, Friedman’s two-way ANOVA test showed that there was a significant effect for the factor stimulation electrode (p = 0.001). No significant effect was observed for the factor modulation frequency (p = 0.807). Post-hoc Wilcoxon signed-rank test with correction for multiple comparisons using the Bonferroni-Holm method (number of comparisons: n = 4) showed that all differences between basal and apical conditions were significant (p = 0.009; p = 0.015; p = 0.019; p = 0.042). For the AMFD data, only the eight subjects who participated in all conditions (see Section "Data collection") were considered in the statistical analysis. The Friedman’s two-way ANOVA test showed no significant effect for the factor stimulation electrode, neither for the 20% frequency change (p = 0.656) nor for the 75% frequency change (p = 0.265). The factor modulation frequency also had no significant effect (p = 0.102 for 20%, p = 0.158 for 75%). Although no statistically significant differences were found between apical and basal stimulation in the AMFD-ACC results, the median absolute N1 amplitude was higher for apical than for basal stimulation in all conditions, except for the difference in the conditions 1 and 5 (10 Hz base frequency, 20% frequency change, basal vs. apical stimulation).

Additionally, for the AMFD task, it was expected that the absolute N1 amplitude would be higher for larger modulation frequency changes. Comparing the 20% with the 75% modulation frequency change in Fig. 4d, it can be seen that this was the case for all conditions except for the comparison of conditions 1 and 2 (basal electrode, 10 Hz base frequency, 20 vs. 75% frequency change). However, the Friedman’s two-way ANOVA test with eight subjects showed no significant effect for the factor frequency change for the basal electrode (p = 0.710). The factor frequency change showed a trend towards significance for the apical electrode (p = 0.063).

**Figure 5:**
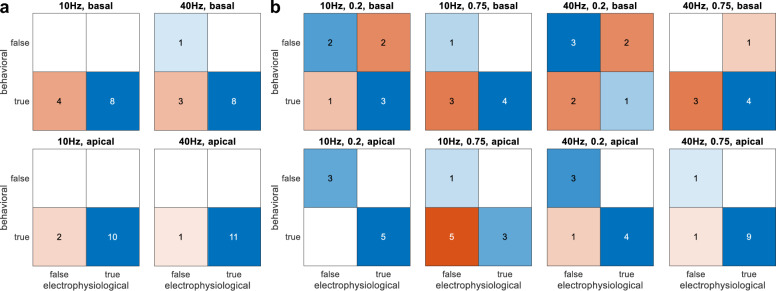
Comparison of behavioral and electrophysiological detection. (**a**) Amplitude modulation detection (AMD); (**b**) Amplitude modulation frequency discrimination (AMFD). Behavioral responses were considered detected (true) if the percentage of correct responses exceeded the chance level (50.3%), otherwise they were considered undetected (false). Electrophysiological responses were considered detected (true) if the N1 peak was significant, otherwise they were considered undetected (false). Responses in which behavioral and electrophysiological detection were in agreement (true positive or true negative) are shown in blue. Responses in which behavioral and electrophysiological detection disagreed (false positive or false negative) are shown in orange. The number indicates the number of subjects. Note that for the 2-stimulus AMFD, not all subjects participated in all test configurations (see Section "Data collection").

To provide a comparison between behavioral and EEG responses, change detection in both modalities was compared in confusion matrices (Fig. 5). Behavioral responses were considered detected if the percentage of correct responses exceeded the chance level (50.3%), as determined by a binomial distribution around the guessing rate (1/3) with a 5% confidence interval. Electrophysiological responses were considered detected if the N1 peak was significant (see Section "Data analysis" for definition of the statistics).

In general, there was a good agreement between EEG and behavioral responses. For AMD, there was a correspondence between EEG and behavioral responses in 79% of the cases (71% basal, 88% apical) whereas for AMFD, the correspondence was 69% (56% basal, 81% apical). However, the EEG detection was less sensitive than the behavioral detection because there were more false negatives (26) than false positives (5). This bias must be taken into account as it may lead to an underestimation of the subject’s performance when analyzed by EEG testing alone.

#### Correlation between behavioral amplitude modulation and speech data

**Figure 6:**
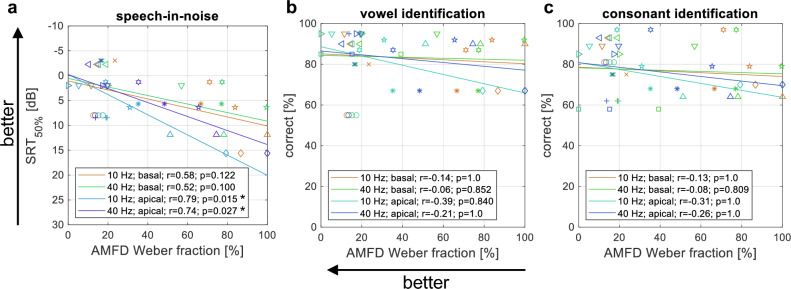
Relationship between behavioral amplitude modulation frequency discrimination (AMFD) and various speech tests. Each subject is represented by a unique marker symbol and the solid line represents the linear fit. The Pearson correlation coefficient r and its p-value after correction for multiple comparisons (number of comparisons: n = 4) are shown in the legend. (**a**) Oldenburg Sentence Test (OLSA) speech reception threshold (SRT_50%_); (**b**) Percent correct vowel identification; (**c**) Percent correct consonant identification. Stars indicate a significant correlation (*p < 0.05).

The fourth hypothesis was that AMFD, as assessed behaviorally in a 3AFC task, would be related to speech recognition, i.e., that a lower Weber fraction would be associated with better speech recognition. The relationship between the behavioral AMFD and the speech recognition is shown in Fig. 6. Note that the behavioral AMD was not related to speech recognition in this study because we only examined the 100% modulation depth, which was easily perceived by most subjects and thus leads to ceiling effects in the analysis. However, other studies have shown that AMD is also correlated with various measures of speech perception^13,14^.

The SRT_50%_ showed a significant linear correlation with the Weber fraction at 10 and 40 Hz with apical stimulation (r = 0.79; p = 0.015 for 10 Hz; r = 0.74; p = 0.027 for 40 Hz). The relationship between SRT_50%_ and the Weber fraction was moderate but not significant at 10 and 40 Hz with basal stimulation (r = 0.58; p = 0.122 for 10 Hz; r = 0.52; p = 0.1 for 40 Hz). No significant correlation between logatome identification scores and speech recognition was observed, but all conditions showed the expected trend that vowel and consonant identification scores were higher with lower Weber fraction. Note that all p-values are given after correction for multiple comparisons using the Bonferroni-Holm method (n = 4).

When the AMFD Weber fraction was averaged between the basal and apical electrodes, the correlation between the averaged Weber fraction and SRT_50%_ was significant for both comparisons, 10 Hz (r = 0.69; p = 0.036) and 40 Hz (r = 0.64; p = 0.033), after Bonferroni-Holm correction (n = 2).

#### Correlations between behavioral speech data and electrophysiological amplitude modulation data

**Figure 7:**
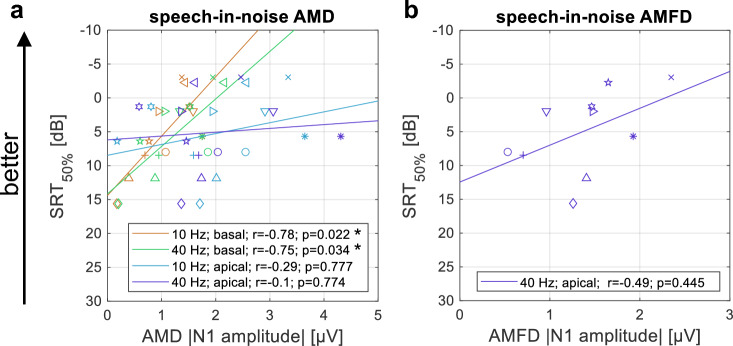
Relationship between the absolute N1 amplitude in the electrophysiological tests and the speech-in-noise speech reception threshold (SRT_50%_). Each subject is represented by a unique marker symbol and the solid line represents the linear fit. The Pearson correlation coefficient r and its p-value after correction for multiple comparisons (number of comparisons: n = 5) are shown in the legend. (**a**) Amplitude modulation detection (AMD); (**b**) Amplitude modulation frequency discrimination (AMFD) for 75% frequency change (condition 8). Stars indicate a significant correlation (*p < 0.05).

The fifth hypothesis was that the ACCs could be used to predict speech recognition across subjects. To reduce the number of correlations to avoid multiple comparisons, we only compared the EEG results with the speech-in-noise results, as the behavioral data did not show a correlation with the vowel or consonant identification scores. The correlation between the SRT_50%_ and the absolute N1 amplitude of all four AMD-ACC conditions is shown in Fig. 7a. The correlation between the SRT_50%_ and the absolute N1 amplitude of the 40 Hz AMFD-ACC with apical stimulation (condition 8) is shown in Fig. 7b. Note that this was the only AMFD-ACC condition in which more than five subjects had significant N1 peaks, and therefore we did not use the other conditions for the evaluation.

In the AMD conditions, the SRT_50%_ correlated significantly with the absolute N1 amplitude at 10 Hz and 40 Hz with basal stimulation (r = -0.78; p = 0.022 for 10 Hz; r = − 0.75; p = 0.034 for 40 Hz). The correlation between SRT_50%_ and the absolute N1 amplitude was weak at 10 Hz with apical stimulation (r = − 0.29; p = 0.777). The responses at 40 Hz with apical stimulation did not correlate with SRT_50%_, but showed the expected trend that speech recognition was better with higher absolute N1 amplitude. In the AMFD condition, the correlation between the absolute N1 amplitude was weak at 40 Hz with apical stimulation (r = -0.49; p = 0.445) but also showed the expected trend. Note that all p-values are given after correction for multiple comparisons using the Bonferroni-Holm method (n = 5).

When the AMD-ACC N1 amplitude was averaged between the basal and apical electrodes, the correlation between averaged N1 amplitude and SRT_50%_ was not significant for both comparisons, 10 Hz (r = − 0.51; p = 0.212) and 40 Hz (r = − 0.41; p = 0.209), after Bonferroni-Holm correction (n = 2).

## Discussion

TEMs are an important feature of speech^3,4^ and CI users rely heavily on TEM encoding to understand speech. In this study, we investigated the relationship between different measures of TEM encoding and speech recognition in CI users, as these measures have the potential to serve as more objective predictors of speech recognition^13–15^ and may also be useful for objective fitting of CI parameters^40–42^. In a novel 3-stimulus EEG paradigm, we were able to simultaneously record CAEPs, ACCs, and ASSRs with direct electrical stimulation of single CI electrodes. We observed correlations between TEM encoding and speech-in-noise recognition. For better clinical applicability, further improvements in the signal quality of ACCs and shortening of the EEG acquisition time are needed.

The novel 3-stimulus EEG paradigm was compared to an alternating 2-stimulus paradigm proposed by Undurraga et al.^28^. The alternating 2-stimulus paradigm^28^ examines AMFD-ACC and ASSR. With our novel 3-stimulus paradigm, we were able to additionally examine AMD-ACC, onset, and offset CAEPs, allowing for a more complete assessment of ERPs and comparisons between the different types of ERPs. However, for AMFD-ACCs, our replication of the alternating 2-stimulus paradigm provided better responses than the novel 3-stimulus paradigm. One reason for this may be that the lower alternation rate of 0.5 Hz in the 2-stimulus paradigm compared to 1 Hz in our 3-stimulus paradigm may lead to less cortical adaptation. Calcus et al.^29^ showed that ACC amplitudes are higher at slow alternation rates, but they only examined alternation rates equal to and higher than 1 Hz. Another reason may be a prolonged cortical adaptation recovery caused by the combination of different ACCs. As shown in the behavioral data (Fig. 1c,d), the AMD task was easier than the AMFD task, resulting in higher AMD-ACC amplitudes compared to AMFD-ACC amplitudes (Fig. 2c,e). It is possible that the high preceding AMD-ACC response in the 3-stimulus paradigm prolonged cortical adaptation recovery, resulting in a smaller subsequent AMFD-ACC amplitude. Further research is needed to assess how cortical adaptation alters the responses in paradigms that combine different ACCs. Finally, the amplitude of the 2-stimulus AMFD-ACC in this study was lower than in the reference study^28^. The differences could be explained by methodological differences between the two studies. The main differences were the EEG systems (ActiveTwo, Biosemi B.V., Netherlands vs. SynAmps 2/RT, Compumedics Ltd., Australia), the stimulation pulse shape (quadra-phasic pulses vs. biphasic pulses), and the examined modulation base frequency (20 Hz vs. 10 Hz and 40 Hz).

We observed an effect of stimulation electrode in the CAEPs, ACCs, and ASSRs. The amplitude for stimulation on an apical electrode was higher compared to a basal electrode. Two effects could contribute to this observation: First, previous studies^20,38,39^ have shown that electrical stimulation on basal electrodes generally generates lower ERP amplitudes than on apical or mid-array electrodes. Possible reasons for this effect could be the longer duration of deafness in basal regions^20^ in combination with the resulting increased neural degeneration^39^ or the greater distance between the electrode and the modiolus^43^. Second, other studies^44–46^ have suggested that TEMs are encoded more strongly by apical than by basal neurons. Since the increased absolute N1 amplitude is observed not only for AMD-ACCs and AMFD-ACCs, but also for the onset and offset CAEPs, we assume that the first effect dominates our results. Additionally, we did not observe improved performance with apical stimulation compared to basal stimulation in the behavioral AMD and AMFD experiments. This is also consistent with other studies that have observed a high variability across stimulation electrodes and no significant differences on behavioral AMD^32,41^ and AMFD^47^ thresholds at different electrode locations. A study by Pfingst et al.^48^ observed a significant deterioration of the behavioral AMD threshold on basal electrodes compared to apical electrodes only at a lower stimulation rate of 250 pps but a significant improvement at a higher stimulation rate of 4000 pps. Because of the variability of ERP amplitudes across stimulation electrodes, it may be important to consider more than just one stimulation electrode when relating single-electrode TEM encoding to the overall performance of CI users. However, averaging the results of basal and apical stimulation did not improve the correlation between speech-in-noise recognition and AMD or AMFD measures in this study.

We investigated AMD and AMFD for two different modulation frequencies: 10 Hz and 40 Hz. The modulation frequencies were chosen in a range that is known to be important for speech recognition^11^. A 40 Hz modulation frequency elicited reliable ASSRs in CI users^35^, and in addition the 40 Hz AMD-ACC has also been associated with speech recognition^30^. Lower modulation frequencies between 4 and 16 Hz (syllabic and sub-syllabic rate) may contribute even more to speech intelligibility^49^, so we also examined TEM encoding at 10 Hz. It would have been interesting to examine even lower modulation frequencies, as frequencies between 4 and 8 Hz have highest power in the speech envelope^50^ and the neural entrainment to theses frequencies is highly related to speech recognition^6,51,52^. However, a long temporal integration time to detect the change in modulation frequency is required for AMFD-ACC^28^, and we suspected that low frequencies of 4 to 8 Hz would not evoke reliable AMFD-ACCs due to the even longer temporal integration time (length of a modulation cycle: 125 ms to 250 ms). We did not observe any significant effect of modulation frequency in the behavioral AMD and AMFD results. There was also no significant effect of modulation frequency on N1amplitude in the electrophysiological results.

We assessed behavioral and electrophysiological AMD and AMFD and compared the results. As expected from previous studies investigating behavioral AMD thresholds^13,14^, almost all subjects were able to detect the 100% perceptual modulation behaviorally. In the behavioral AMFD task, we observed a high variability in the Weber fraction, which is also consistent with other studies^14,34^. The highest modulation frequency change analyzed was 75%, the same as used by Undurraga et al.^28^, and it was discriminated by most subjects. Comparing the behavioral and electrophysiological results of this study was challenging because the target measures were different. Behaviorally, we estimated the number of correct responses for the AMD and AMFD conditions, whereas the electrophysiological target was the absolute N1 amplitude. Although the ACC amplitude is related to the degree of change^26^, it may not be directly comparable to the number of correct responses or the Weber fraction. In addition, we were limited by ceiling effects in the behavioral analysis of AMD. Confusion matrices comparing behavioral detection or discrimination with the presence/absence of an ACC response per se showed moderate to strong agreement between the behavioral and electrophysiological results (79% for AMD and 69% for AMFD). However, the electrophysiological detection of significant ACC was less sensitive than the behavioral detection. A similar problem was reported by Mao et al.^18^, who reported an overestimation of DR when the T-level was estimated from EEG amplitude growth functions compared to behavioral responses. In addition, electroencephalography may be less sensitive than behavioral detection because of the multiple algorithms required to clean the data from different types of artifacts, which cannot be excluded to reduce ERP responses.

One important aspect of this study was to investigate the relationship between TEM encoding and speech recognition. Previous studies have shown that behavioral AMD^13,14^ and AMFD^14^ thresholds correlate with speech recognition in CI users^13,14^, and that behavioral AMFD thresholds measured shortly after CI surgery can predict 6-month speech recognition scores^15^. This study complements the state-of-the-art, by demonstrating a significant correlation between behavioral AMFD thresholds and SRT_50%_ in experienced CI users at two different modulation frequencies. One advantage of AMFD is that low AMFD thresholds are associated not only with good TEM encoding, but also with the ability to accurately detect amplitude rise times^53^. Amplitude rise time detection is associated with better phase locking or neural entrainment, which in turn improves speech recognition^50^. The low SNR of AMFD-ACC in both the 2-stimulus and the 3-stimulus paradigms resulted in high variability in N1 amplitudes that were not significantly correlated with speech recognition. Further improvement of the paradigm and increased AMFD-ACC SNR are needed. The N1 amplitude of the AMD-ACC at 10 Hz and at 40 Hz was correlated with SRT_50%_, which is consistent with Han and Dimitrijevic^30^ who showed a correlation between the N1 latency and various measures of speech recognition, including word perception in noise. In this study, we did not observe any correlation between TEM encoding and logatome identification tests. One reason for this finding could be the overall high logatome identification scores, which showed little variability between subjects.

In general, this study included a wide range of subjects varying in age, etiology of deafness, and onset of profound hearing loss. Variability in these and other factors affects the individual CI speech recognition performance^7,8^. For example, the factor age could lead to cognitive decline or temporal processing deficits that could corrupt TEM encoding^54^. In addition, age could affect the cortical responses, but the extent to which this is the case is still debated in the literature^54^. In children the N1 peak of the CAEP is known to be less reliable and to develop throughout childhood^55,56^. Therefore, it could be problematic to apply this study in children, although they would benefit most from a more objective assessment of hearing performance. Further research is needed to investigate whether the ACC amplitude is equally affected by subject variability. If this is the case, we suggest compensating for subject variability by examining the amplitude of the ACC relative to the amplitude of the onset CAEP (e.g., (|Amplitude_ACC_|-|Amplitude_CAEP_|)/|Amplitude_CAEP_|). The 3-stimulus paradigm examined in this study, or a combination of the 2-stimulus paradigm and the recording of onset CAEPs, would allow for such a relative assessment, and we will explore the potential of such a relative measure in future analyses.

EEG recordings in CI users are often challenging due to electrical artifact. In this study, we used a clinically certified EEG system with a 20 kOhm sampling rate, a CI pulse rate of 500 pps, and state-of-the-art CI artifact rejection strategies. We used a high-density EEG cap, but also showed that it is still feasible to measure ERPs using a single recording channel (FCz, mastoid, ground), as would be preferred in the clinic. The advantage of EEG over behavioral tests is that no active participation of the patient is required and attention has been shown to have no significant effect on N1^57^, making EEG more objective than behavioral tests. A major drawback of the current implementation is the long recording time required to obtain reliable responses (~ 1 ½ hours to test all conditions in the 3-stimulus paradigm). Further research is needed to reduce the recording time while improving the recording quality. One method of reducing recording time is to use higher rates of alternation between the stimuli, as suggested by Calcus et al.^29^, which could reduce recording time by a factor of three. Another method to reduce recording time is, to focus on only the most relevant conditions, e.g., only on a single modulation frequency and a single modulation frequency change, which would reduce recording time by a factor of four. One method to improve signal quality would be to use invasive EEG electrodes that are located closer to the cortical signal generators and are less affected by artifacts, as shown by Haumann et al.^58^. Invasive electrodes could include epidural electrodes implanted during CI surgery^58^ or electrodes from the CI, which have recently been shown to be suitable for continuous recording of cortical potentials^59,60^. The results can be used to predict speech recognition in patients who cannot participate in behavioral speech recognition tasks, and at the same time the results can be used to improve the fitting of CI parameters. For example, deactivating electrodes^40,41^ or increasing the C-level^42^ on electrodes with high AMD thresholds has been shown to improve speech-in-noise recognition. TEM encoding abilities can also be used to compare performance with different stimulation rates or strategies, which could help to select the most appropriate strategy for each patient^15^. Finally, CAEPs have been shown to correlate with T-level at the level of individual CI electrodes, so that assessment of CAEP amplitude growth functions may replace eCAPs and eABRs as objective predictors of T-level^18,20^.

In conclusion, the electrophysiological assessment of TEM encoding is a promising method for a more objective assessment of speech recognition in CI users. In addition, the results of electrophysiological testing can be linked to the fitting of CI parameters, bringing CI technology one step closer to a closed-loop CI. The present study shows that it is possible to simultaneously measure CAEPs, ACCs and ASSR in a novel 3-stimulus paradigm with a clinical EEG system, and that the responses are related to behavioral TEM encoding and speech-in-noise recognition results. The ideas and results of this study may pave the way for new clinically applicable methods to assess TEM encoding and speech recognition in CI users based on EEG.

## Methods

### Subjects

Twelve CI users participated in the study (8 male, 4 female; median age: 64 years; range: 40–82 years). Participant demographics are summarized in Supplementary Table A1. Participants were implanted with a unilateral or bilateral Nucleus CI (Cochlear Ltd., Australia). All participants had fully functional devices with a fully inserted electrode array and self-reported no neurological or psychiatric disorders. All participants had at least two years of CI experience. Written informed consent was obtained from all participants prior to the experiment. The study was carried out in accordance with the Declaration of Helsinki principles and approved by the Ethics Committee of the Hannover Medical School.

### Data collection

All subjects were invited to three study appointments. At the first appointment, the subjects underwent a fitting procedure, speech tests, a loudness calibration, a loudness balancing and psychoacoustic evaluations. At the second appointment, we measured EEG with a novel 3-stimulus paradigm. At the third appointment, we measured the EEG with a 2-stimulus paradigm. In the 2-stimulus paradigm, we measured only condition 8 in subject 1531 and subject 5766, and only conditions 6 and 8 in subject 2889 due to time constraints. Subject 8111 could not participate in the third appointment due to health conditions. The parameters that were used for the different conditions are summarized in Supplementary Table A2 and individual conditions for each subject are summarized in Supplementary Table A1.

#### Fitting procedure

First, a clinical CI fitting procedure was performed using the Custom Sound 6.0 software (Cochlear Ltd., Australia) for best comparability between subjects. Threshold level (T-level) and comfort level (C-level) were determined for each CI electrode. Equal loudness across electrodes was ensured by presenting four consecutive pulse trains on four adjacent electrodes, and the subject was asked to balance the loudness of the last pulse trains with respect to the previous three pulse trains. Next, the four consecutive pulse trains were shifted apically by one electrodes and the same procedure was repeated until all electrodes were loudness balanced. A CP910 sound processor (Cochlear Ltd., Australia) with the stimulation parameters described in Stimulation Parameters was used, and all automatic front-end algorithms such as SCAN or noise reduction were deactivated.

#### Speech tests

Vowel and consonant identification was assessed using the MACarena application^61^, which is based on the German Minimal Auditory Capability (MAC) battery^62^. For the vowel identification task we used eight different vowels that were presented in consonant–vowel (CV) logatomes (“Da”, “De”, “Di”, “Do”, “Du”, “Dö”, “Dä”, “Dü”) in quiet. The CV logatomes were presented in a random order and the subject was asked to select the correct CV logatome from a list of all CV logatomes. During 20 repetitions of training, feedback was provided to indicate correct or incorrect responses. The actual test included 40 repetitions without feedback. The vowel identification score was defined as the percentage of correct responses. For the consonant identification task, we used twelve different consonants that were presented in vowel-consonant–vowel (VCV) logatomes (“aPa”, “aTa”, “aKa”, “aBa”, “aDa”, “aGa”, “aFa”, “aSa”, “aLa”, “aRa”, “aMa”, “aNa”) in quiet. The test procedure was the same as for CV, with 24 repetitions for training and 48 repetitions for the actual test.

Speech-in-noise recognition was investigated using the German OLSA. The test consists of five-word sentences, such as “Stefan nahm zwölf kleine Ringe”, presented at a fixed noise level. An OLSA list contains 20 of these sentences. The SNR was automatically adjusted based on the subject’s performance, converging on the SNR at which 50% of the words were correctly understood. This SNR is referred to as the SRT_50%_. Subjects performed two lists for training and two lists for testing. The final speech-in-noise test score was the average SRT_50%_ of the two test lists. The lists were randomized across subjects.

All speech stimuli were presented from a single loudspeaker in front of the subject. Listening with the ear contralateral to the tested ear was prevented by using in-the-ear and on-the-ear protectors and by removing the CI processor or, in the case of bimodal subjects, the hearing aid. The presentation level for all stimuli was 65 dB SPL(A), pre-calibrated with an XL2 sound level meter (NTi AUDIO, Switzerland). In the OLSA, the noise level was 65 dB SPL(A), while the signal level was automatically adjusted during the adaptive procedure. The subjects performed the speech tests using a research processor with the individual clinical map created at the beginning of the session.

#### Stimulation parameters

All psychoacoustic and electrophysiological evaluations were performed with direct stimulation using the research interface Nucleus Implant Communicator (NIC, version 4.3.0), a CP910 sound processor, a Programming Pod (all Cochlear Ltd., Australia), and a computer with the Matlab interface (version 2022b). The stimuli consisted of anodic-phase-first biphasic pulses. The pulses had a phase width of 25 µs, an interphase gap of 8 µs and were presented at a stimulation rate of 500 pps. This relatively low stimulation rate was used to allow removal of the CI artifact by linear interpolation between artifact-free intervals^27,35^. The stimulation mode was always monopolar (MP1 + 2).

**Figure 8:**
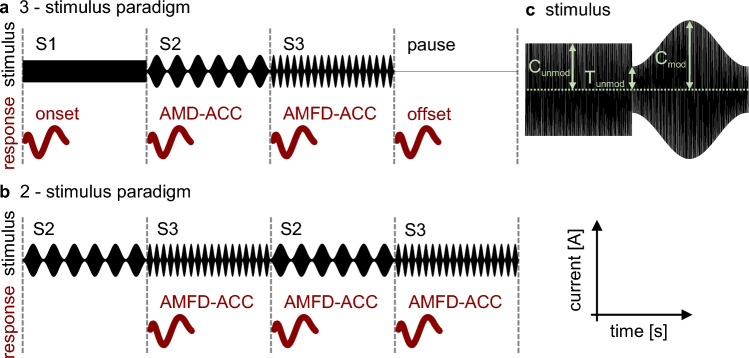
Schematic illustration of the stimuli. (**a**) 3-stimulus paradigm with the stimulus in black and the expected neural response in red. The transitions between the stimuli (S1, S2, S3, pause) were expected to evoke the onset response, the acoustic change complex (ACC) related to amplitude modulation detection (AMD), the ACC related to amplitude modulation frequency discrimination (AMFD), and the offset response; (**b**) 2-stimulus paradigm with the stimulus in black and the expected neural response in red. The transition between the alternation stimuli (S2, S3) was expected to evoke an ACC related to AMFD; (**c**) Levels of the unmodulated stimulus (C_unmod_, T_unmod_) and the modulated stimulus (C_mod_, T_unmod_).

An overview of the different stimulation parameters is given in Supplementary Table A2, and a schematic representation of the stimuli and their expected neural responses for the different EEG paradigms is shown in Fig. 8. The stimulation electrodes were electrode 3 (except for subjects 2541 and 0828 it was electrode 4 because electrode 3 was deactivated) and electrode 20. These electrodes were chosen to represent a basal and an apical region of the cochlea, respectively. Stimulus S1 was an unmodulated pulse train presented at the level C_unmod_. S2 was an amplitude modulated pulse train that was modulated between C_mod_ and T_unmod_ at the base frequencies (F_S2_, i.e., 10 and 40 Hz). S3 was another amplitude modulated pulse train that was modulated between the same C_mod_ and T_unmod_ at deviant frequencies (F_S3_). F_S3_ was specified as a frequency increment with respect to the base frequency such that (F_S3_-F_S2_)/F_S2_ was 20% and 75% (i.e., 12 Hz, 17.5 Hz for 10 Hz and 48 Hz, 70 Hz for 40 Hz).

In all amplitude modulation experiments, we used 100% perceptual amplitude modulation, meaning that the amplitude was fully modulated between the individual threshold (T_unmod_) and the comfort (C_mod_) level. Note that the term modulation refers to an amplitude modulation in current domain, i.e., the current-level unit (CL) was translated to actual current in ampere, which was then modulated and translated back to CL.

#### Loudness calibration and balancing

Loudness levels were more precisely determined by the following procedure: The subject was presented with the 500 ms long unmodulated stimuli (see Supplementary Table A2, S1), and was asked to rate the loudness on a ten-point scale (0 = no stimulation, 6 = comfortable, 10 = too loud). The subject was asked to adjust the level so that the loudness was first at the upper comfort level (UC-level, transition from 7 to 8), second at C-level (6), and third at T-level (transition from 1 to 0). The levels were set by the subject using the up and down arrows on a keyboard, where each key press corresponded to an increment/decrement of one CL. This procedure was repeated three times, and the average levels were defined as the final levels. The calibrated C-levels and T-levels are referred to as C_unmod_ and T_unmod_. In the loudness balancing procedure that followed, we made sure that the UC-level was never exceeded.

McKay and Henshall^63^ showed that loudness cues confound the perception of amplitude modulated with respect to unmodulated pulse trains in CI users. Therefore, we balanced the perceived loudness between unmodulated and amplitude modulated stimuli (see Supplementary Table A2, S1 and S2), following their procedure. The C-levels for amplitude modulated stimuli are referred to as C_mod_. Loudness balancing between different modulation frequencies was not performed because previous studies^33,34^ have shown that the loudness of low-frequency amplitude modulated pulse trains is almost constant across modulation frequencies.

#### Psychoacoustic measure

AMD and AMFD were studied using the method of constant stimuli in a three-alternative forced-choice (3AFC) paradigm. We presented three 0.5 s long stimuli, with a 0.5 s pause between stimuli, the same durations as used by Undurraga et al.^28^. Please note that the stimuli were shorter than the stimuli that were used in the EEG paradigm. However, it has been shown that neither AMD nor AMFD thresholds are affected by stimulus duration, as long as the stimuli are longer than the temporal integration window of 5 modulation cycles^64^, which corresponds to 500 ms for 10 Hz AM. For AMD, we investigated whether the subject was able to perceive the difference between the unmodulated stimulus (S1) and the amplitude modulated stimulus (S2). For AMFD, we tested whether the subject was able to perceive the difference in modulation frequency. In this task, two amplitude modulated stimuli were presented: S2 and S3. Besides the eight conditions defined in Supplementary Table A2, additional frequency increments were analyzed such that (F_S3_-F_S2_)/F_S2_ was 10%, 20%, 40% and 75%. In both tasks, the subject was asked to identify the stimulus that was different from the other two stimuli. The task was repeated 25 times for each condition and the percentage of correctly identified stimuli was calculated. The order of the three stimuli and the order of the conditions was randomized across subjects.

In the AMFD task the just noticeable difference (JND) between modulation frequencies F_S2_ and F_S3_ was calculated from the behavioral responses. The JND is defined as the middle point of the psychometric function^65^. For this purpose, we fitted a sigmoidal function between the guessing rate (33%) and the lapsing rate (100%) and estimated the JND at 66% correct responses. The Weber fraction (expressed as a percentage) was calculated as the ratio between the JND_66%_ and the base frequency F_S2_. The Weber fraction provides a measure of AMFD that is comparable across different modulation frequencies^34^.

#### Electroencephalographic measure

The EEG recordings were performed using a SynAmps 2/RT clinical biosignal amplifier with the high-density 64-channel EEG Quik-Cap. The cap consisted of 64 passive silver/silver chloride sintered electrodes that were arranged according to the extended 10/20 system, with a ground electrode at the AFz position. We added a reference electrode on the nose, four electrodes for vertical and horizontal bipolar electrooculogram (VEOG, HEOG), which were used for eye artifact suppression, and two mastoid electrodes (M1, M2). The EEG was recorded using Curry 9 software (all Compumedics Ltd., Australia). The trigger output of the CI Programming Pod was connected to the SynAmps connector box, to allow synchronization of EEG and stimuli. Subjects washed their hair prior to the study, and the electrode-to-skin impedances were maintained below 20 kOhm using ECI electrolyte gel (Electro-Cap International, Ohio, US). During the recording in an electromagnetically and acoustically shielded chamber, subjects were asked to sit calm and relaxed while watching a silent movie with subtitles.

##### 3-Stimulus paradigm

In this electrophysiological measurement we combined the AMD and AMFD task by presenting sequences of three different stimuli without pause (see Fig. 8a): Stimulus S1, S2 and S3. The transition from S1 to S2 was intended to evoke an AMD-ACC at the change from an unmodulated to a fully modulated pulse train. The transition from S2 to S3 was intended to evoke an AMFD-ACC at the change between modulation frequencies. In addition to the two ACCs at the transitions between stimuli, the paradigm allowed us to analyze the responses at the onset of S1 and at the offset of S3, as well as the continuous ASSR during the presentation of S2 and S3. The length of each stimulus was at least 1 s and corresponded to a multiple of the modulation cycle. The sequence of three stimuli was followed by a 1 s silent interval. The change between stimuli coincided with the minima of a modulation cycle. The combination of the three stimuli resulted in eight different conditions (see Supplementary Table A2). The eight conditions were repeated 315 times, so that for each parameter combination there were 1260 trials for onset and offset CAEPs, 630 trials for AMD-ACCs, and 315 trials for AMFD-ACCs. The conditions were randomized across repetitions and subjects. Subjects were allowed to take a break every 150 repetitions (~ 30 min) or on demand.

##### 2-Stimulus paradigm

The ACC amplitudes of the 3-stimulus-paradigm, especially for the AMFD-ACC, were small compared to the responses measured by Undurraga et al.^28^. Therefore, we decided to replicate the stimulus pattern of Undurraga et al.^28^ by presenting S2 and S3 in an alternating order (see Fig. 8** b**). The transition from S2 to S3 and vice versa was intended to evoke an AMFD-ACC. S2 and S3 each lasted ~ 2 s (corresponding with a multiple of the modulation cycle), as in the reference study, and the change between stimuli coincided with the minima of a modulation cycle. The alternation was repeated 160 times, so that for each parameter combination there were 319 trials for AMFD-ACC. In addition to the eight conditions summarized in Supplementary Table A2, we also measured a ninth sham condition with a 0 CL stimulation, which resulted in no sound perception by the subject but transmitted power through the RF link of the CI. The order of presentation of the nine conditions was randomized across subjects. Subjects were allowed to take a short break after each condition (~ 10 min) and a longer break every three conditions.

### Data analysis

Raw EEG data were imported into Matlab (version 2022b) using the EEGLAB^66^ library (version 2021.1) with the “loadcurry” plugin (version 3.2.3). The stimulus artifact was removed using the blanking method^35^. For this purpose, the signal at the expected stimulus pulse positions was replaced by a linearly interpolated signal. Linear interpolation was performed between the last pre-stimulus sample and the 1.9 ms post-stimulus sample^33^. Other studies have shown the functionality of this artifact rejection approach for CI evoked ASSRs^33,67^ and ACCs^28,68^. Supplementary Fig. A3 shows the EEG before and after linear interpolation. The data were downsampled to 500 Hz using a zero-phase Kaiser window FIR filter for anti-aliasing. The data were filtered with a Hamming window sinc FIR filter using a low pass with a cutoff frequency of 100 Hz and a high pass with a cutoff frequency of 1 Hz in succession. The data was stored in a bdf file format for further processing.

The bdf files were reloaded and processed in Python (version 3.9) using the “pEEGy-python”^28^ toolbox (version 1.1.8). Data were re-referenced to the mastoid electrode contralateral to the stimulation side (M1 or M2). We did not use the common average reference or the mean of both mastoids reference to avoid introducing additional CI artifact from highly contaminated electrodes near the CI to all other electrodes. Channels with an impedance greater than 20 kOhm and automatically detected noisy channels with a standard deviation greater than three times the average standard deviation of all channels were discarded. Following the pipeline of Undurraga et al.^28^, a template matching algorithm^69^ using the data from the two additional EOG channels (VEOG and HEOG) was used to remove blink artifacts. The data were epoched into trials based on the trigger signal and sorted into different stimulus conditions. Each trial was 1 s in the 3-stimulus paradigm and 2 s in the 2-stimulus paradigm. Spatial filtering based on denoising source separation (DSS) was performed to remove stimulus-unrelated artifacts. Details about DSS are described elsewhere^70,71^, but basically the signal is divided into orthogonal components through multiple steps of normalization and principal component analysis (PCA) rotation. The components are ordered by “evoked-to-total power ratio”^71^. This means that the first DSS components contain stimulus-related signal, such as the ERP or CI artifact, and the last DSS components contain stimulus-unrelated signal, such as motion artifact and other noise sources. Undurraga et al.^68^ have shown that the combination of linear interpolation and DSS is an effective artifact removal method with minimal distortion for ACC and minimal phase distortion for ASSR.

For the onset and offset responses and the ACCs, only the first 20 DSS components were retained. The EEG was reconstructed based on these first 20 DSS components, primarily including the ERP and CI artifact. We did not explicitly remove manually selected CI artifact components because sometimes these components contained a mixture of an ERP and CI artifact and we wanted to avoid diminishing the ERP by removing these components. The data were again low-pass filtered using a zero-phase Kaiser window FIR filter with a cutoff frequency of 30 Hz to reduce residual CI artifact. It should be noted that while this filter completely removed the CI artifact for S1 in all conditions and for S2 and S3 in the 40 Hz base frequency conditions, it cannot be excluded that some CI artifact remained in S2 and S3 in the 10 Hz base frequency conditions. However, this residual artifact cannot be confused with an ERP because the artifact was continuous throughout the stimulation period. Baseline correction was performed by subtracting the time average of each trial. The weighted average^72,73^ of the trials was calculated, whereby blocks of five trials where weighted with their normalized inverse variance across trials. N1 peaks were automatically detected in the time window from 70 to 200 ms relative to the stimulus change. The SNR of the peak was calculated in dB as the power of the peak amplitude divided by the power of the residual noise (RN) of all epochs, whereby RN represented the variance of trials and time averaged across the whole trial. The peak was classified as significant, when the peak amplitude exceeded 2∙RN. The results of the ERP analyses (including peak amplitudes, peak latencies, peak significance, SNR, and averaged waveforms) were collected in an SQLite database along with the results of the behavioral tests (including speech-in-noise scores and vowel and consonant identification scores).

For ASSR, we discarded DSS components higher than 20, and additionally we discarded DSS components that contained CI stimulation artifact. The DSS components were visually detected and removed when they showed a constant oscillation over the stimulus interval and had high power at the CI stimulation side. On average, we manually removed one additional DSS component per subject. Supplementary Fig. A3 shows example topographies with and without this CI artifact rejection with DSS. The Fast Fourier Transform was used to calculate the complex spectrum of each channel in each epoch, and the magnitudes were averaged across epochs. The SNR was calculated in dB as the power of the spectral magnitude of the frequency peak divided by the power of the RN of all epochs, whereby RN represented the variance of magnitudes averaged across adjacent frequencies (plus/minus 5 Hz of the peak frequency). The results of the ASSR analyses (including peak amplitudes and SNRs) were also collected in an SQLite database.

### Supplementary Information

Supplementary Information.

## Data Availability

Raw data generated as part of this work will be made available upon reasonable request. Please contact the corresponding author.
