# Supplementary material for: Psychoacoustic and electroencephalographic responses to changes in amplitude modulation depth and frequency in relation to speech recognition in cochlear implantees
Source: Sci Rep. 2024 Apr 8;14:8181. doi: 10.1038/s41598-024-58225-1 (PMC11002021; doi:10.1038/s41598-024-58225-1)
Supplement: Supplementary file 1 — Supplementary Information. [file 41598_2024_58225_MOESM1_ESM.pdf]

Supplementary Information

Psychoacoustic and electroencephalographic responses to changes in amplitude modulation depth and frequency in relation to speech recognition in cochlear implantees

Nina Aldag[1]* and Waldo Nogueira[1]

[1]Department of Otolaryngology, Hannover Medical School and Cluster of Excellence ‘Hearing4all’, Hanover, Germany

*corresponding author (contact: aldag.nina(at)mh-hannover.de)

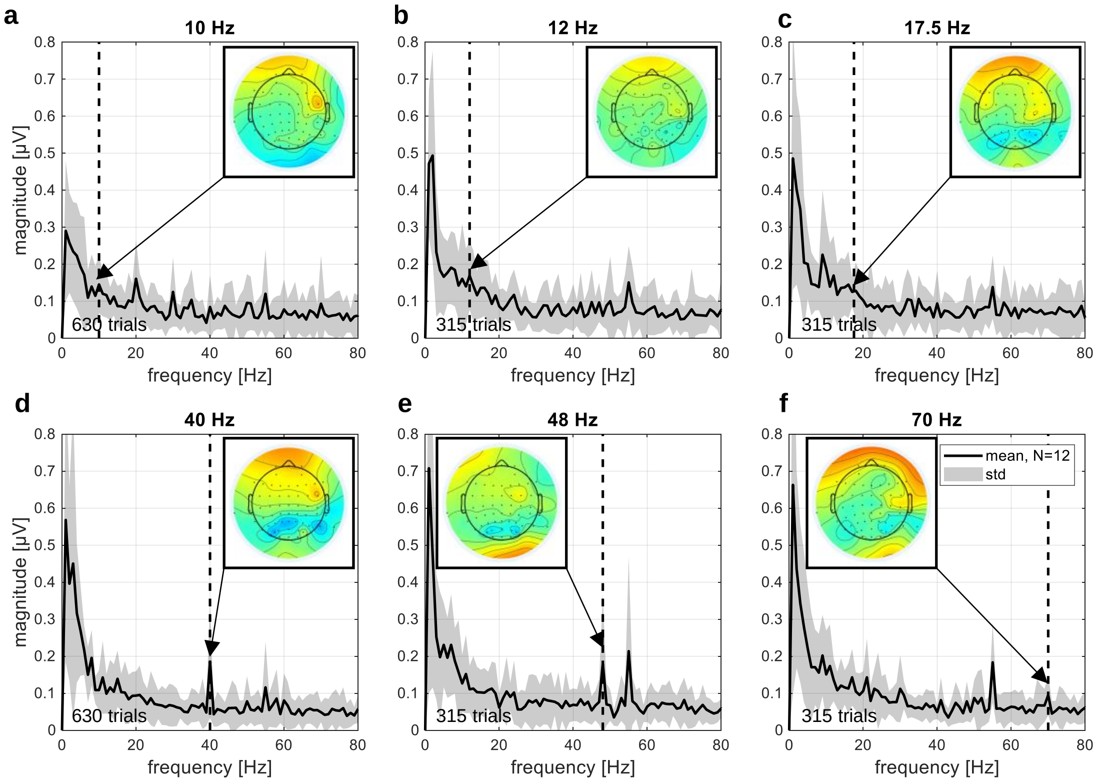

Figure A1: Auditory steady-state response (ASSR) for stimulation with a basal electrode at the modulation frequencies **(a)** 10 Hz; **(b)** 12 Hz; **(c)** 17.5 Hz; **(d)** 40 Hz; **(e)** 48 Hz; **(f)** 70 Hz at the electrode position FCz. The mean magnitude across subjects is shown in bold black and individual magnitudes are shown in gray. The dotted vertical line marks the modulation frequency of the stimulus. Topographic plots of the ASSR show the average voltage spread across all subjects at different electrode locations in the axial plane, with the nose at the top. The relative power in dB is color-coded, with red representing the highest power and blue representing the lowest power.

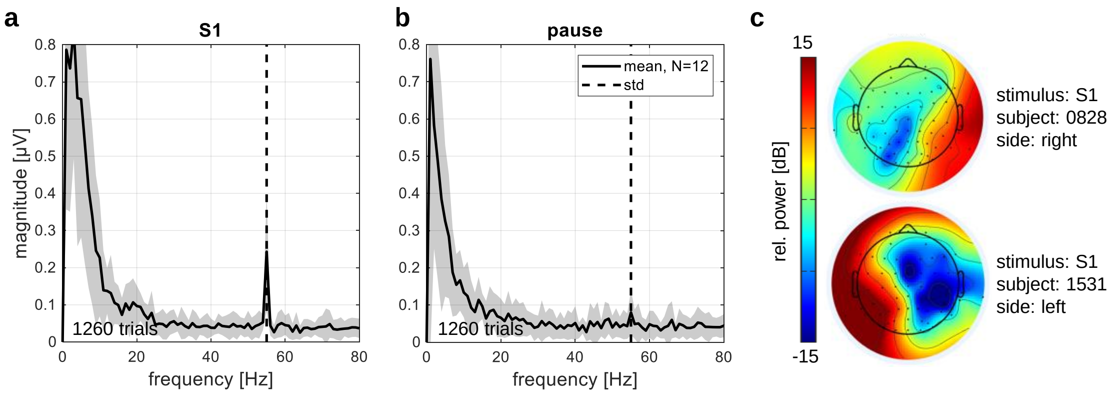

Figure A2: 55 Hz artifact signal during **(a)** the unmodulated stimulus (S1) and during **(b)** the pause, both at electrode position FCz. The mean magnitude across subjects is shown in bold black and individual magnitudes are shown in gray. The dotted vertical line marks the 55 Hz artifact frequency. The topographic maps **(c)** show the average voltage distribution at different electrode positions in the axial plane, with the nose at the top. The relative power in dB is color-coded. The maps are shown for one subject with the active implant on the right (subject 0828) and one subject with the active implant on the left (subject 1531). The stimulation side is associated with the highest power, indicating that the artifact is produced by the cochlear implant.

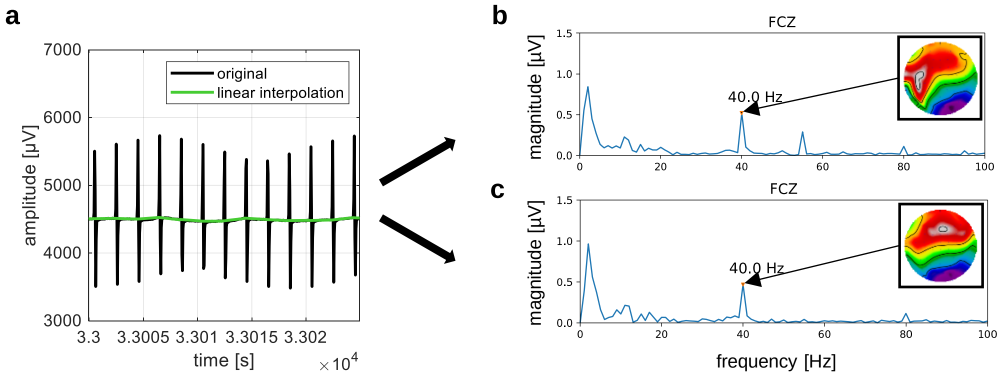

Figure A3: Artifact rejection: **(a)** Amplitude over time before (black) and after (green) linear interpolation; **(b)** Frequency distribution without DSS artifact rejection of example subject 4977 with the CI on the left ear. The topographic map shows the average power distribution at 40 Hz in the axial plane, with the nose at the top. The relative power in dB is color coded. The highest power was located near the left ear; **(c)** Magnitude of different frequencies after DSS artifact rejection. The highest power was located in the fronto-central region, and the magnitude of the 40 Hz peak was slightly reduced compared to panel **(b)**.

Table A1: Overview of demographics and measurement conditions of cochlear implant (CI) users. Parameter details about the measurement conditions can be found in **Supplementary Table A2**. F = female; M = male; R = right; L = left.

Subject	Age [years]	Gender	Etiology of Deafness	Test Ear	CI Electrode Array	Time of Deafness	CI experience [years]	Modality	CI Electrode		Conditions	
									Basal	Apical	2-Stim	3-Stim
0275	82	F	Unknown	R	CI522	postlingual	6	bimodal	3	20	1-8	1-8
0828	78	M	Sudden hearing loss	R	CI512	postlingual	13	bilateral	4	20	1-8	1-8
1038	54	M	Meningitis	L	CI24RE (CA)	perilingual	10	bilateral	3	20	1-8	1-8
1531	75	M	Unknown/ stress	L	CI24RE (CA)	postlingual	9	bilateral	3	20	8	1-8
1926	70	M	Otosclerosis cochleae	L	CI24RE (CA)	postlingual	13	bilateral	3	20	1-8	1-8
2541	52	F	Unknown	L	CI422	prelingual	11	bilateral	4	20	1-8	1-8
2889	66	M	Unknown/ stress	R	CI624	postlingual	2	bilateral	3	20	6, 8	1-8
4479	74	M	Unknown	L	CI522	postlingual	7	bilateral	3	20	1-8	1-8
5766	40	M	Sudden hearing loss	L	CI512	postlingual	11	bilateral	3	20	8	1-8
7129	49	F	Unknown	R	CI24RE (CA)	prelingual	18	bilateral	3	20	1-8	1-8
8111	43	F	Rubella embryopathy	R	CI24RE (CA)	prelingual	9	bilateral	3	20	-	1-8
9205	62	M	Unknown	R	CI624	postlingual	11	bilateral	3	20	1-8	1-8

Table A2: Summary of stimulation parameters of all stimuli (S1-S3) in the different conditions. The modulation frequency F_{S3} represents a frequency increment of 20 % and 75 % with respect to F_{S2}. MD = modulation depth; F_{SX} = modulation frequency.

		S1		S2		S3	
Condition	Electrode	MD [%]	F_{S1} [Hz]	MD [%]	F_{S2} [Hz]	MD [%]	F_{S3} [Hz]
1	basal	0	N/A	100	10	100	12
2							17.5
3					40		48
4							70
5	apical				10		12
6							17.5
7					40		48
8							70
